# Artificial Intelligence in Inflammatory Bowel Disease Endoscopy: Advanced Development and New Horizons

**DOI:** 10.1155/2023/3228832

**Published:** 2023-04-17

**Authors:** Yu Chang, Zhi Wang, Hai-Bo Sun, Yu-Qin Li, Tong-Yu Tang

**Affiliations:** Department of Gastroenterology, First Hospital of Jilin University, Changchun, 130000 Jilin, China

## Abstract

Inflammatory bowel disease (IBD) is a complex chronic immune disease with two subtypes: Crohn's disease and ulcerative colitis. Considering the differences in pathogenesis, etiology, clinical presentation, and response to therapy among patients, gastroenterologists mainly rely on endoscopy to diagnose and treat IBD during clinical practice. However, as exemplified by the increasingly comprehensive ulcerative colitis endoscopic scoring system, the endoscopic diagnosis, evaluation, and treatment of IBD still rely on the subjective manipulation and judgment of endoscopists. In recent years, the use of artificial intelligence (AI) has grown substantially in various medical fields, and an increasing number of studies have investigated the use of this emerging technology in the field of gastroenterology. Clinical applications of AI have focused on IBD pathogenesis, etiology, diagnosis, and patient prognosis. Large-scale datasets offer tremendous utility in the development of novel tools to address the unmet clinical and practice needs for treating patients with IBD. However, significant differences among AI methodologies, datasets, and clinical findings limit the incorporation of AI technology into clinical practice. In this review, we discuss practical AI applications in the diagnosis of IBD via gastroenteroscopy and speculate regarding a future in which AI technology provides value for the diagnosis and treatment of IBD patients.

## 1. Introduction

Artificial intelligence (AI) represents the capacity of machines to imitate human intelligence. Major aspects of AI applications in medicine include computational intelligence, gene sequencing, intelligent diagnosis, and medical robotics. Currently, the application of AI technology to gastrointestinal endoscopy is increasing rapidly. Compared with professional endoscopists, AI technology has been found to have superior accuracy for analyzing and processing large volumes of medical data. Machine learning (ML), which is essential in the implementation of AI, is the process of using algorithms to guide a computer to use known data to obtain an appropriate model. This model can then be used to assess new situations. As a branch of contemporary statistics, ML is particularly useful for analyzing complex data [1]. There are four types of ML: supervised learning, unsupervised learning, semi-supervised learning, and reinforcement learning. In supervised learning, an algorithm is given a dataset that includes questions and correct answers, and the machine learns how to predict the correct answers to future questions by analyzing the data. Deep learning (DL) is a subset of ML [2] that has received attention in the field of medical imaging science as a fully automated, fast, and accurate imaging analysis solution. A typical example of DL is the convolutional neural network (CNN), which can be used to address image-based problems in medicine. Given their great utility, CNNs have become widely applied in medical imaging [2].

Inflammatory bowel disease (IBD) is a chronic complex inflammatory disease with increasing incidence globally. IBD prognosis is closely related to the healthcare system [3]. The two subtypes of IBD, ulcerative colitis (UC) and Crohn's disease (CD), are typical complex diseases characterized by chronic and heterogeneous presentations. They are induced by interactions between genomic, environmental, microbial, and immunological factors [4]. The accurate diagnosis of IBD has long been a challenge for gastroenterologists. However, new IBD diagnostic techniques include combinations of methods such as gastrointestinal endoscopy, molecular pathology, epigenetics, metabolomics, and proteomics [5]. The state-of-the-art endoscopic imaging techniques and novel biomarkers provide new approaches to the differential diagnosis of IBD. Over the past few years, the importance of endoscopy in the diagnosis, treatment, and monitoring of IBD has been established. For example, dye-chromo endoscopy (DCE) and virtual chromo endoscopy (VCE) are often used in the endoscopic surveillance of IBD [6]. In DCE, topical dye is sprayed on the colon wall, enhancing the visualization of mucosal morphology. This technique is now recognized as the gold standard for diagnosing hyperplasia and has been included in the recommendations of international diagnosis guidelines. VCE, which is also included in international guidelines, involves the digitization of endoscopic images, enabling tissue surface details to be enhanced with high accuracy. Accordingly, it can function as an alternative to DCE. Despite the utility of these techniques, differences in the specific methods used as well as the quality and subsequent interpretation of diagnostic components can significantly affect the diagnosis and treatment outcomes of IBD among gastroenterologists.

Today, progress in AI technology has dramatically enhanced the ability of clinicians and researchers to analyze, manipulate, interpret, and apply large data sets. The amount of data from clinical trials, medical imaging, and genetic research (genomic, transcriptomic, and proteomic) is rapidly increasing [7]. Without appropriate methods for interpretation, it is difficult to combine large amounts of clinical data with genetic data for detailed analysis in clinical practice. AI and ML can be used to quickly analyze these datasets, enabling clinicians to implement stratified management of patients in terms of risk assessment, diagnosis, treatment, and prognosis. Thus, AI and ML have enabled more accurate and standardized endoscopic treatment measures. With the enhanced application of AI in IBD treatment and diagnosis, endoscopic procedures have become increasingly specialized and currently include the evaluation of endoscopic disease activity, monitoring of cancerous lesions, and capsule endoscopy (CE) for the diagnosis of CD [8]. The purpose of this review is to summarize the current application of AI techniques to the diagnosis and treatment response prediction of IBD and to discuss future directions in the applications of AI to IBD endoscopy.

## 2. Classification of AI

As a popular research topic, interest in AI has grown rapidly in the medical community over recent years. Accordingly, the application of AI to IBD treatment has led to significant progress in computer-assisted diagnosis and therapy [9]. Many computer algorithms have been developed to assist in gastroscopy. ML, as a form of AI, can be used to facilitate algorithmic self-improvement based on experience and without human supervision. Specifically, the ML algorithm learns from inputted data sets, and identifies behavior patterns or generates predictive models [10]. The application of ML in endoscopic IBD monitoring can be realized by analyzing still images. DL, as a subset of ML, can be used to handle complicated learning algorithms. CNNs, as a type of DL, are becoming the leading technology for image processing.

### 2.1. Machine Learning

In the near future, ML-based models are expected to be employed by a large number of clinicians for image recognition and analysis. In the field of IBD research, ML has been used to determine whether the mucosa is healing in patients with UC [11] and to classify subtypes of pediatric colonic IBD [12]. Huang et al. invented a computer-aided diagnostic system based on ML and DL (DLML-CAD). The concept underlying the system is related to transfer learning, in which a classifier is trained to extract the desired features of images using a network that has been pre-trained using millions of non-medical images. The investigators chose the deep neural networks(DNN), support vector machine, and k-nearest neighbor network models as classifiers for the DLML-CAD, and classified hundreds of images as Mayo endoscopic subscore (MES) 0–1 or MES 2–3. The DLML-CAD has reached or even slightly surpassed the diagnostic level of IBD endoscopists. The system can identify and analyze colon endoscopic images to accurately determine the degree of mucosal healing (ML). It can also be used to evaluate the mucosa in different areas of the colon during colonoscopies in patients with UC [11]. Dhaliwal et al. collected clinical, endoscopic, radiographic, and histological data from 74 patients with colonic IBD, trained a random forest classifier on the complete dataset, and used ML to identify three histological features and four endoscopic examinations that could be used to distinguish colonic UC from CD [12]. According to the existing data, the ML model performs very well in the field of IBD diagnostics and treatment. However, its widespread application in clinical settings is still uncertain as it is still in the clinical trial stage.

### 2.2. Deep Learning

Given the rigorous training required to accurately assess endoscopic inflammation and the limited number of endoscopists with specialized training, DL may offer many advantages in the clinical evaluation of IBD patients. According to a relevant study, small bowel ulcers in patients with CD, as revealed via video CE, can be effectively monitored by DL models when the area under the receiver operating characteristic curve is between 0.94 and 0.99 [13]. Takenaka et al. also validated the use of DL algorithms for assessing disease activity using a UC endoscopic severity score [14], and DL algorithms appear to be of trans-generational significance for the rapid acquisition and analysis of images during endoscopy.

### 2.3. Convolutional Neural Networks

CNNs are a type of DL algorithm that have an enormous impact on the field of computer vision, with image analysis accuracy comparable to that of professional physicians. CNNs simulate neuronal networks in the brain by combining many data inputs, weights, and biases. These systems are extremely useful in detecting intestinal ulcers, erosions, and strictures [15, 16]. Ding et al. validated the ability of a CNN-based ML model to efficiently identify and analyze small bowel capsule endoscopic images (SB-CE) and concluded that it could be a powerful tool to help experienced endoscopists quickly and accurately analyze and classify small bowel lesions [17], whereas Klang et al. monitored endoscopic ulcers in video capsule endoscopic images and successfully identified CD patients via an AI system based on CNNs [13]. However, no algorithms have performed in a superior manner to professional endoscopists. Furthermore, AI algorithms for the long-term monitoring of IBD patients have not yet been developed. Many such models are in the clinical trial stage and, thus, are not widely used in large-scale clinical practice.

## 3. AI in the Diagnosis of IBD

The diagnosis of IBD is a highly sophisticated process, because it must be carefully discriminated from other diseases. Clinicians must consider the patient's history and clinical presentation, and conduct a series of endoscopic and histological examinations. Endoscopic techniques are the cornerstone of IBD diagnosis, and endoscopists use a variety of methods to classify and analyze mucosal erosions, inflammation, and ulcers. These include histological examinations such as microscopic biopsy combined with a series of imaging examinations such as Computed Tomography (CT) and Magnetic Resonance Imaging (MRI). The endoscopic diagnosis of IBD requires highly specialized operators with extensive training and experience. However, inter-operator variability in endoscopic evaluation is inevitable. Such variation may be addressed by AI-based computer-assisted systems.

### 3.1. AI in Diagnosing UC

As endoscopic remission is a therapeutic goal for UC patients, endoscopy has been widely used to assess the activity and efficacy of treatments [18]. Histological remission has also become an increasingly important therapeutic goal for UC [19]. However, to determine the status of histological remission targets, pathologists must evaluate mucosal inflammation. This can result in observer variability and, thus, data heterogeneity. Computer-aided systems can help physicians to determine the degree of inflammation and ML during UC endoscopy, resulting in a more accurate assessment of histological remission.

One study demonstrated that the predictions of a model based on DL algorithms regarding the severity of UC during endoscopy were largely consistent with those of experienced human evaluators [20]. Thus, such algorithms may improve the assessment and treatment of UC according to endoscopic data. Sutton et al. investigated the use of DL algorithms in differentiating UC from other intestinal diseases and evaluating the severity of UC endoscopic ulcers [21]. They used a dataset containing 851 images of UC patients that had been labeled and graded by professional endoscopists using the MES. Other studies on the automatic grading of UC endoscopic images have also shown advantages of DL models. For instance, Takenaka et al. evaluated endoscopic images of UC patients using a model based on a DL network [14]. Their model was highly accurate (90.1%) when evaluating endoscopic images of 40,758 UC patients with endoscopic remission who had received a UC Endoscopic Severity Index Score (UCEIS) of 0. Similarly, Yao et al. modified the traditional full-motion video operation model by segmenting the endoscopic video into 1-frame-per-second image stacks and then automatically rotating, fragmenting, and pre-processing the images to conform to a standard scale [22]. The resulting model was able to automatically generate MESs for patients. Gottlieb et al. utilized CNNs to collate 795 full-length endoscopic videos, and collected and cleaned the data so that the endoscopic Mayo score and the UCEIS scores could be used for prediction and analysis [23]. To reduce the subjectivity of operator-assessed endoscopic image activity scoring, Bossuyt et al. developed a non-operator-dependent objective endoscopic scoring system to assess UC disease activity. The system was based on the red, green, and blue pixel values and endoscopic image recognition, and it performed UC activity assessment with high operability and objectivity [24]. More details can be shown clearly in Table 1.

The DNN model for UC assessment can score endoscopic images with very high accuracy. Indeed, many studies have shown that DNNs can assess the activity and remission of intestinal mucosal inflammation through endoscopic images alone, eliminating the need for biopsies and reducing the need for pathology. The objectivity and consistency of such systems are comparable to that of professional endoscopists. However, many challenges must be addressed before AI can be formally applied to large-scale clinical practice. For example, the data used to train DNNs are defined, organized, and structured by humans based on collected images, which can lead to problems such as the human assessment of discrepancies.

### 3.2. AI in Diagnosing CD

Because the intestinal inflammatory permeation in CD includes the small intestine, especially the terminal ileum, CE is often used to detect CD when the applications of conventional colonoscopy are limited. However, CE can generate videos that are 8–10 hours in length, which makes frame-by-frame inspection and analysis very time-consuming and labor-intensive for endoscopists [17]. In response to this, a number of CNN algorithms have been developed.

In 2019, Ding et al. validated the ability of CNN-based algorithms to analyze SB-CE images with greater accuracy and sensitivity than conventional methods and reported a significantly reduced reading time compared with routine analysis by gastroenterologists [17]. Indeed, this technique has substantial benefits for gastroenterologists in terms of efficiency and convenience in organizing and analyzing image information. In 2019, Aoki et al. developed a CNN-based algorithm that could automatically detect erosions and ulcers from capsule endoscopic images. They tested their model using 10,440 test images and found the sensitivity, specificity, and accuracy to be 88.2%, 90.9%, and 90.8%, respectively [25]. In 2019, Klang et al. designed and trained a CNN to randomly segment 17,640 capsule endoscopic CD images [13]. They achieved good results with an area under the curve of 0.99 and an accuracy of 95.4–96.7%. In 2021, Klang et al. further evaluated a CNN-based AI system. They explored the accuracy of the system in comparing CD stenosis and different degrees of ulcers and found that such systems may be capable of automatic identification and grading of CD in the near future [26]. In 2021, Barash et al. successfully developed a DL algorithm for CE that could automatically grade CD ulcers, demonstrating that CNN-assisted CE readings have high utility in the diagnoses and monitoring of CD patients [27]. More details can be shown clearly in Table 2.

Given the findings mentioned above, AI-based DL algorithms, especially CNNs, appear to strongly improve the accuracy of CE analysis while greatly reducing the time required for endoscopists to examine images and videos. However, these DL-based detection algorithms are performed at the level of individual images but not at the entire video level. This means that the samples for these experiments are from retrospective studies and do not fully resemble the performance of video CE. Future researchers working with AI technologies are likely to develop algorithms that can automatically evaluate CE videos, resulting in accurate scoring systems that can be widely used in clinical practice. As the technology continues to advance, the combination of AI and CE is expected to significantly impact endoscopy. In the future, AI-assisted CE may be able to perform rapid and systematic examinations of entire intestinal lesions in less than 30 minutes. In the meantime, CE systems that facilitate patient diagnosis, treatment, and biopsy appear to be driving a revolution in endoscopy technology.

### 3.3. AI in Cancer Surveillance of IBD

Given that IBD is a long-term chronic condition, patients have a greatly increased risk of colorectal cancer compared with the general population. One study revealed that the existence of low-grade dysplasia (LGD) in the intestine of IBD patients served as a high-risk factor for progressing to high-grade dysplasia (HGD) or even colorectal cancer. Therefore, once LGD is detected, patients should undergo careful endoscopic screening [28]. In IBD patients, endoscopic screening for colorectal cancer, LGD, and HGD is currently performed mainly via stained endoscopy plus endoscopic resection or biopsy [29]. To date, no AI systems have been developed for the long-term monitoring of patients with IBD colitis. In 2020, Maeda et al. reported the first case of AI-assisted detection of colitis-associated neoplasms [30]. Their patient was 72 years old and possessed an 18-year history of colitis. In 2021, Maeda et al. designed an automated AI algorithm-based polypectomy surveillance system for use during surveillance colonoscopy. Their system was able to clearly and efficiently identify colonic lesions in non-IBD patients, which confirmed the feasibility of the system for helping non-endoscopic specialists identify and detect long-term heterogeneous growths in IBD patients.

## 4. AI in the Treatment of IBD

Histological remission has gradually become a therapeutic goal for UC, replacing previous goals such as endoscopic and symptomatic remission. Accurate assessments of the degree of histologic remission and intestinal inflammatory activity may allow for more precise and efficient treatments as well as reduce the need for multiple repeated gastrointestinal examinations [9]. Unfortunately, clinical practice is currently lacking a simple and easy standard for evaluating histological remission. In 2022, Villanacci et al. developed a semi-supervised AI inductive transfer learning system consisting of two modules. Their goal was to apply the simplified neutrophil-only Paddington International virtual ChromoendoScopy ScOre histological remission index (PHRI) developed and validated by pathologists to a computer-aided diagnostic system. When comparing the evaluation results produced by the AI with those generated by pathologists, the AI model was found to be highly sensitive and specific in determining the presence of neutrophils, indicating that it could be an excellent support in determining whether patients had achieved histological remission [31]. Gui et al. also developed a CNN-based computer-aided UC histological diagnosis and scoring system for identifying disease activity in UC patients based on PHRI item scores. Their system was designed to not only avoid the subjectivity of pathologists in determining the degree of inflammatory activity but also to reduce the degree of difficulty of judgments regarding patient status. The PHRI is a simple and reproducible scoring system that is well suited for large-scale applications in clinical practice. It is effective in assessing UC endoscopic activity and also allows physicians to make more accurate conclusions about the status of histological remission [32]. However, although the studies reviewed above proposed using the PHRI score as a histological diagnostic and grading tool for UC, their work contains certain limitations. For example, in the study by Gui et al., the follow-up protocol excluded endoscopic and histological reassessment, the duration of the follow-up period was relatively short, and the investigators did not calculate overall PHRI scores for different regions of the intestine.

Given the complexity and chronicity of IBD as an immune intestinal disease, as well as the multiple factors that influence patient outcomes, the goal of many current therapies is symptomatic relief and ML. To this end, various targeted drugs and biological agents have been used in clinical practice. Maintaining the integrity of the intestinal epithelial barrier has become a research hotspot and an emerging therapeutic target in the biomedical field. Sahoo et al. used an ML approach to build a network that identified a pathway rich in gene clusters that maintain intestinal epithelial barrier integrity, leading to the identification of a top intestinal barrier protector for the treatment of IBD [33]. Experts in the field are expected to use similar methods to invent additional drugs and therapeutics that maintain or repair the intestinal epithelial barrier, and this is likely to become an exciting research area.

For many years, minimally invasive techniques have been used to treat IBD. Currently, robotic surgery is becoming an efficient and precise complement to, and potentially a future replacement for, minimally invasive surgery. There is growing evidence indicating that surgical robots have significant advantages over minimally invasive techniques such as laparoscopic surgery, including better patient safety, reduced surgical complications, and shortened prognosis [34]. In 2020, Hota et al. investigated the differences in perioperative and treatment outcomes between open, laparoscopic, and robotic surgeries for treating CD [35]. They selected a database containing data from 5,158 patients with CD, utilized Convolutional point transformer (CPT) codes to determine the procedures used for patient ileal resection, compared the incidence of anastomotic fistula between the three surgical approaches, and applied multivariate analysis to derive a 95% confidence interval for the dominance ratio. They found that robotic surgery was a non-inferior treatment for both colonic resection in UC recipients and ileostomy in CD patients [34, 35].

## 5. Risks and New Horizons

AI applications focused on disease prediction and cancer surveillance, and the diagnosis and treatment of IBD have been found to be extremely reliable and efficient. In the future, AI technologies may be able to completely replace endoscopists for decision-making and treatment, or alternatively, endoscopists may act as assistants to AI systems. For this to occur, prerequisites for the large-scale use of AI algorithms in clinical practice must be met, and ethical guidelines regarding patient safety must be put in place. Endoscopy clinics and academies will also need to develop emergency measures for malfunctions during AI treatment and remedies for treatment errors. In addition to the above, the biases from developers of AI-based algorithms must be considered, as most datasets are human-trained. Finally, the impact of different dataset types and analyses must be compared to accurately predict the status and value of AI algorithms in IBD clinical practice.

These above-mentioned risk factors should not prevent us from continuing to research AI algorithms, refine system functionality, and work to realize the full potential of AI-assisted medicine. The positive impact of AI and DL in gastroenterology is substantial, and many publicly available datasets are available for further comparison and analysis by researchers. In terms of future development, new AI algorithms for long-term monitoring of colorectal cancer in IBD patients are urgently needed to improve the prediction of cancer risk and time-course of treatment. Furthermore, randomized controlled trials are necessary to investigate the benefits and feasibility of using AI in the clinical management of IBD patients compared with general clinical management, especially in terms of variations in treatment measures, outcomes, and treatment costs. Currently, endoscopists are collaborating with algorithm developers with the goal of using large data sets for medical imaging AI. Indeed, the creation of data sets for collecting and training AI to capture new types of images will require the expertise and experience of endoscopists.

## 6. Conclusions

This review summarizes recent applications of AI in the endoscopic examination and treatment of IBD and predicts possible future directions for the use of AI in treating patients with IBD. We expect that AI will soon become a cornerstone of endoscopy and IBD treatment. Besides, it will be necessary to translate the large amount of current exploratory data into evidence that can be applied to clinical practice before AI could be widely used in clinical settings. This will require not only the rapid development of AI but also the cooperation and commitment of endoscopists, specialists, and societies.

## Figures and Tables

**Table 1:** Endoscopic AI applications in diagnosing UC.

Author	Year	Data source	Purpose	Results
Sutton et al.	2022	851 images of UC patients	To differentiate UC from other intestinal diseases and to evaluate the severity of UC endoscopic ulcers	The accuracy (87.50%) and area under the curve (AUC, 0.90)
Takenaka et al.	2020	40,758 images of endoscopies and 6,885 biopsy outcomes of 2012 UC patients	To create a deep neural network system for analyzing endoscopic images of UC patients	The remission in endoscopy with 90.1% accuracy (95% and 89.2–90.9%)
Najarian et al.	2021	Video of the clinical trial set with 51 high resolution and 264 tests	To trial a fully automated video system for analyzing and grading endoscopic disease in UC	Automated Mayo endoscopic subscores (MES) scoring of clinical trial videos correctly differentiated between remission and active disease in 83.7% of cases
Gottlieb et al.	2021	795 full-length endoscopy videos of 249 patients	To verify a deep learning, algorithm can be trained to predict levels of UC severity from full-length endoscopy videos	(0.787–0.901) for endoscopic Mayo Score (eMS) and 0.855 (95% confidence interval, 0.80–0.91) for UCEIS
Bossuyt et al.	2020	29 consecutive patients with UC and 6 healthy controls	To develop an operator- independent computer-based tool to determine UC activity based on endoscopic images	RD correlated with Robarts histological index (RHI) (*r* = 0.74, *p* < 0.0001), MES (*r* = 0.76, *p* < 0.0001), and UC endoscopic index of severity scores (*r* = 0.74, *p* < 0.0001)

**Table 2:** Endoscopic AI applications in diagnosing CD.

Author	Year	Data source	Purpose	Results
Ding et al.	2019	4,206 abnormalities in 3,280 patients	To analyze SB-CE images with greater accuracy and sensitivity than conventional methods	99.88% sensitivity in the per-patient analysis (95% confidence interval [CI], 99.67–99.96)
Aoki et al.	2019	113,426,569 images from 6,970 patients	To develop a CNN-based algorithm that could automatically detect erosions and ulcers from capsule endoscopic images	With 99.88% sensitivity in the per-patient analysis (95% CI, 99.67–99.96) and 99.90% sensitivity in the per-lesion analysis (95% CI, 99.74–99.97)
Klang et al.	2019	17,640 CE images from 49 patients	To evaluate a deep learning algorithm for the automated detection of small-bowel ulcers in Crohn's disease (CD) on capsule endoscopy (CE) images	AUCs (area under the curve of 0.99 and accuracies ranging from 95.4–96.7%
Klang et al.	2021	27,892 CE images	To prove the ability of deep neural networks to identify intestinal strictures on CE images of Crohn's disease (CD) patients	A differentiation between strictures and normal mucosa (area under the curve [AUC], 0.989)
Barash et al.	2021	17,640 CE images from 49 patients	To develop a deep learning algorithm for automated grading of CD ulcers on CE	The accuracy of the algorithm was 0.91 (95% CI) for distinction of 3 different grades
